# Fluid resuscitation in the critically ill: what is the next challenge?

**DOI:** 10.5935/0103-507X.20150053

**Published:** 2015

**Authors:** Naomi Hammond, Simon Finfer

**Affiliations:** 1 Critical Care and Trauma Division, The George Institute for Global Health - Sydney, Australia.; 2 Malcolm Fisher Department of Intensive Care Medicine, Royal North Shore Hospital - Sydney, Australia.; 3 Sydney Medical School, University of Sydney - Sydney, Australia.

## Fluid resuscitation in the critically ill: What are the remaining challenges?

In 2010 the SAFE-TRIPS investigators reported on resuscitation fluid administration in 391 intensive care units (ICUs) in 25 countries.^(1)^ The study found that more than one third of patients in the ICUs received resuscitation fluids on the study day and that the choice of fluid administered was determined by local practice and habit rather than by any identifiable patient characteristic. Given that many thousands of patients receive resuscitation fluid each day, it is an intervention that has the potential to result in either great benefit or great harm. Although recently published trials by clinician investigators have dramatically increased the evidence base in this area,^(2-9)^ some questions remain unanswered.

## What do we know?

Published in 2004, the Saline versus Albumin Fluid Evaluation (SAFE) Study was the first ICU "mega-trial".^(7)^ The SAFE study compared the safety and efficacy of 0.9% saline and 4% albumin for resuscitation in 6997 adult general intensive care patients and established that overall use of the two fluids resulted in almost identical mortality rates and no significant difference in other outcomes. However, in the subgroup of patients with traumatic brain injury, albumin administration increased mortality.^(8)^ Additionally, the SAFE study supported the hypothesis that albumin might decrease mortality in patients with severe sepsis;^(9)^ this observation led to further trials of albumin in patients with severe sepsis and septic shock, unfortunately these trials have not provided a definitive answer.^(3)^

Hydroxyethyl starches (HES) is the other colloid that has been extensively investigated in randomised controlled trials (RCTs).^(2,5,6)^ These RCTs have provided convincing evidence that both higher molecular weight preparations and the newer low molecular weight starches cause harm.^(2,5,6)^ In high quality trials HES administration has consistently increased mortality and the incidence of acute kidney injury and its use results in more patients being treated with renal replacement therapy; these adverse effects are observed both in patients with severe sepsis^(6)^ and in the general ICU population.^(5)^ Other colloids, specifically dextrans and gelatins have not been extensively studied. The Cochrane Collaboration regularly reviews the totality of evidence regarding fluid choice and concludes that colloids offer no demonstrable clinical benefit over crystalloids; colloids are associated with no improvement in survival and are more costly, making their use in clinical practice hard to justify.^(10)^

## Crystalloids are recommended as the first choice for fluid resuscitation but which one should I use?

Normal (0.9%) saline has been the most commonly used resuscitation fluid worldwide^(1)^ despite concerns that its high chloride content is associated with worse patient outcomes.^(11)^ In observational studies, the use of fluids with lower chloride concentrations, such as balanced (buffered) salt solutions, has been associated with reductions in major surgical complications, in the incidence of acute kidney injury, and reductions in hospital mortality.^(11)^ These observational data appear to be influencing clinical practice with increasing use of balanced salt solutions in some regions.^(12)^ Large scale RCTs comparing outcomes in patients assigned to receive either normal saline or balanced salt solutions are currently being planned (NHMRC APP1101765).

## I know what fluid to use but how much should I give?

Another remaining challenge is deciding whether a liberal or restrictive fluid practice is best for critically ill patients. A positive fluid balance is associated with adverse patient outcomes in patients with sepsis and in patients with renal failure.^(13)^ Although this is likely confounded by severity of illness with sicker patients being more likely to have a positive fluid balance, it begs the question of whether we should re-evaluate the liberal use of resuscitation fluids in ICUs. Recent trials suggest that adopting a more restrictive fluid strategy in patients with lung injury and following major abdominal surgery may produce better short term outcomes.^(14,15)^ In another context, the Fluid Expansion as Supportive Therapy (FEAST) trial,^(4)^ reported that African children with severe infections who received fluid boluses (albumin or 0.9% saline) had increased mortality compared with those who did not receive fluid boluses. While the applicability of these results to other healthcare settings is unclear, the impact of fluid boluses and liberal resuscitation strategies should both be studied in large high quality RCTs. Such studies should examine both short and long term outcomes as later cognitive impairment may be more common in patients assigned to a restrictive fluid strategy.^(16)^

## Conclusions

It is clear that "crystalloid or colloid" is the wrong question with irrefutable evidence that different colloid solutions have different effects and the effects are also different in different populations. The same may be true of crystalloids but overall, the existing evidence favours the use of crystalloids as first line resuscitation solutions.

Currently, the two outstanding questions to be addressed are whether chloride restriction through the use of balanced salt solutions and separately fluid restrictive strategies are beneficial to critically ill patients or not. Given the widespread use of resuscitation fluids both these testable hypotheses should be addressed as a matter of public health priority and ultimately for the good of our patients.
